# Frequency of fetal anomalies from pregnant rats with mild diabetes submitted to moderate intensity exercise program

**DOI:** 10.1186/1758-5996-7-S1-A241

**Published:** 2015-11-11

**Authors:** Thalita Bohnen Carneiro, Rafaianne Queiroz de Moraes Souza, Thaigra de Sousa Soares, Thais Leal Silva, Mário Cazar Fiuza, Cristielly Maria Barbosa, Vanessa Caruline Araujo, Kleber Eduardo de Campos, Débora Cristina Damasceno, Gustavo Tadeu Volpato

**Affiliations:** 1 Universidade Federal De Mato Grosso, Barra do Garças, Brazil

## Background

The practice of exercise for diabetes control is common, also during pregnancy. However, its potential benefits and risks of exercise during pregnancy, complicated or not by diabetes, is unknown.

## Objective

To evaluate the fetal anomaly frequency from mild diabetic pregnant rats submitted of exercise of moderate intensity.

## Materials and methods

The experimental severe diabetes was induced in newborn female Wistar rats in the first day of birth by intravenous injection of Streptozotocin in a dose of 100 mg/Kg. In adult life (110 days) the rats were submitted to oral glucose tolerance test (OGTT) to confirm the moderate diabetes. After its confirmation, rats were mated and randomly assigned to 4 experimental groups (minimum n=13 animals/group): Control: non-diabetic pregnant rats without exercise (sedentary); Exercise: non-diabetic pregnant rats exposed to exercise; Diabetic: diabetic pregnant rats without exercise; Diabetic Exercise: diabetic pregnant rats exposed to exercise. The moderate intensity exercise program was swimming, from the 7th to 20th days of pregnancy. At day 21 of pregnancy, the rats were anesthetized and the uterus was removed. Fetuses were analyzed to external, visceral and skeletal anomalies. Analysis of variance followed by Tukey's test was used to mean values and Fisher Exact test was used to proportions. Differences were considered statistically significant when p<0.05.

## Results

There are no changes in ossification sites and external anomalies among the groups. The total number of skeletal abnormalities increased significantly in all experimental groups compared to control group. The exercise associated to diabetes increased the total number of fetuses with skeletal anomalies compared to sedentary diabetic animals. Regarding the visceral anomalies, there was not increase in total number frequency among the groups. There were increase in hidroureter and hydronephrosis in diabetic groups compared to control group.

## Conclusion

The mild diabetes associated to exercise during pregnancy led the increasing in anomalies, showing the exercise practice during gestational period require carefulness.

